# Impact-Resistant Poly(3-Hydroxybutyrate)/Poly(ε-Caprolactone)-Based Materials, through Reactive Melt Processing, for Compression-Molding and 3D-Printing Applications

**DOI:** 10.3390/ma15228233

**Published:** 2022-11-19

**Authors:** Fouad Laoutid, Hadrien Lenoir, Adriana Molins Santaeularia, Antoniya Toncheva, Tim Schouw, Philippe Dubois

**Affiliations:** Polymeric and Composite Materials Unit, Materia Nova Research Center, University of Mons, Nicolas Copernic 3, 7000 Mons, Belgium

**Keywords:** reactive extrusion, polyhydroxyalkanoates, biopolymers, blends, additive manufacturing

## Abstract

Biobased and biocompatible polymers, such as polyhydroxyalkanoates (PHAs), are of great interest for a large range of applications in the spirit of green chemistry and upcoming reuse and recycling strategies. Polyhydroxybutyrate (PHB), as a promising biocompatible polymer belonging to PHAs, is subject to increased research concern regarding the high degree of crystallinity and brittle behavior of the resulting materials. Therefore, the improvement of PHB’s physico-mechanical properties aims to decrease the Young’s modulus values and to increase the ductility of samples. Here, we proposed an ambitious approach to develop melt-processed materials, while combining PHB characteristics with the ductile properties of poly(ε-caprolactone) (PCL). In order to compatibilize the poorly miscible PHB/PCL blends, dicumyl peroxide (DCP) was used as a free-radical promotor of polyester interchain reactions via the reaction extrusion process. The resulting PHB/PCL-DCP materials revealed a slight increase in the elongation at break, and significant improvement in the impact resistance (7.2 kJ.m^−2^) as compared to PHB. Additional decrease in the Young’s modulus values was achieved by incorporating low molecular polyethylene glycol (PEG) as a plasticizer, leading to an important improvement in the impact resistance (15 kJ.m^−2^). Successful 3D printing using fused deposition melting (FDM) of the resulting PHB/PCL-based blends for the design of a prosthetic finger demonstrated the great potential of the proposed approach for the development of next-generation biomaterials.

## 1. Introduction

Since the first synthesis of poly(3-hydroxybutyrate) (PHB) in 1927 by French biologist Maurice Lemoigne, this polymer has been of particular scientific interest due to its biobased, biocompatible, and biodegradable properties [1]. In fact, many bacteria can accumulate crystalline PHB-based granules [2], used as energetic resources when confronted with nutrient stress or when their carbon/nitrogen environment ratio is high, as they do with starch and glycogen. PHB, moreover, is a part of a broad polymer family called poly(3-hydroxyalcanoates) (PHAs), known to find applications in various fields, especially in the medical sector, such as tissue engineering, bio-implants, drug delivery [3], adhesives, and packaging [4] as a result of recent progress in PHA production and modification.

Despite these advantages, PHB remains a highly fragile polymer with very low plastic deformation [5], mainly due to its high degree of crystallinity (over 55%) related to its high macromolecular chain stereoregularity and its physical ageing inducing secondary crystallization [6,7]. This polymer is also characterized by low nucleation density and the ability to slowly crystallize to form large spherulites [8]. On the other hand, the high crystallinity rate favors the fabrication of materials with high stiffness (Young’s modulus values around 2 GPa and tensile strength values around 40 MPa), but also inducing fragility among the material, leading to low elongation at break and low impact resistance. Therefore, such brittle and stiff behavior limits PHB practical use in a large range of applications [9]. Moreover, the degradation temperature (Td) of PHB is relatively close to its melting temperature (Tm), responsible for a narrow melt processing window [10], making its transformation with conventional melt-processing equipment (e.g., extruders, injection/compression molding, and 3D printing equipment) difficult. Even today, the replacement of commonly used polymers by PHAs remains a great challenge that can be overcome by modifying PHAs through biological, chemical and/or physical modifications or combinations thereof [11,12].

Blending PHB with other biopolymers presents a very interesting way of enhancing its properties and reducing the cost of the final materials. The advantage of such approach is the fact that the properties of the final material can be fine-tuned by selecting the suitable polymers, thus adjusting the blend composition and processing conditions. Several studies investigated the effect of blending PHAs with natural or synthetic biopolymers, such as starch, poly(ε-caprolactone) (PCL), polylactide (PLA), polybutylene adipate terephthalate (PBAT) as a way of reducing the cost of the final material while maintaining PHA characteristics such as biodegradability and biocompatibility [12]. However, the incorporation of such biopolymers in PHAs did not show high efficiency for enhancing the mechanical properties of the resulting blends as a result of lack of compatibility between the two polymer phases and very poor interface adhesion [13,14,15]. Various strategies have been approached to improve the compatibility between the biopolymers, such as in situ generation of grafted copolymers at the interface, the addition of compatibilizing agents or plasticizers [15,16,17,18].

The main objective of the present study is to enhance the mechanical properties of PHB through the joint action of PCL as a minor biopolymeric phase and polyethylene glycol (PEG) as a plasticizer. To succeed in this, a reactive melt processing approach is proposed using dicumyl peroxide (DCP) as a free-radical initiator inducing in situ compatibilization via grafting (and possibly cross-linking) reactions between the polymer phases. PCL, as a semi-crystalline aliphatic polyester characterized by a low glass transition temperature (Tg of ca. −60°C), was selected to reduce PHB stiffness owing to its good mechanical properties (PCL has a Young’s modulus around 450 MPa, high elongation at break—500% and tensile strength of 15 MPa [19,20]), in addition to the biodegradable and biocompatible nature of the polyester [18,21]. Low-molecular-weight PEG was proposed for its good miscibility with PHB [22] and its plasticizing effect, well reported in various biopolyesters such as PLA and PBAT [23].

## 2. Materials and Methods

### 2.1. Materials

The materials used in the present study were poly(3-hydroxybutyrate) (PHB) Enmat Y3000p (Mw = 280,000 g.mol^−1^,ρ = 1.25 g.cm^−3^), provided by Tianan Biologic Material Co. Ltd., Ningbo, China, poly(ε-caprolactone) (PCL, CAPA 6500) (Mw = 50,000 g.mol^−1^), ρ = 1.1 g.cm^−3^) and PCL (CAPA 6800, Mw = 80,000 g.mol^−1^), provided by Perstorp UK Limited (Warrington, UK), polyethylene glycol (PEG) provided by Merck (Rahway, NJ, USA) (Mw = 1000 g.mol^−1^), ρ = 1.2 g.cm^−3^) and dicumyl peroxide (DCP, purity 99%) provided by Acros Organics (Geel, Belgium).

### 2.2. Processing

Melt compounding of the different blends was performed in an internal mixer Brabender Plastograph (Buisburg, Germany) at 190 °C for 8 min (3 min mixing at 30 rpm followed by 5 min at 100 rpm). DCP was introduced after 2 min 30 s, and PEG after 3 min, to provide better accessibility to melted polymers. Before processing, all pellets were dried in a ventilated oven (24 h at 40 °C and 60 °C for PCL and PHB, respectively). The obtained blends were then compression-molded at 190 °C using a Agila PE20 hydraulic press (Menen, Belgium) to obtain plates of 8 × 10 × 0.5 cm^3^. More precisely, the materials were first pressed at low pressure (10 bar) for 3 min (five degassing cycles), followed by a high-pressure cycle at 150 bar for 3 min. Specimens for impact and tensile mechanical testing were prepared by milling (using a Carbide Motion 3D milling machine, Torrance, CA, USA) the obtained plates. The composition of the blends is presented in Table 1.

The optimized PHB/PCL-DCP-PEG blends were used to produce filaments (diameter 1.75 mm) suitable for 3D printing and printed using a Prusa 3D printer (Prusa Research, Prague, Czech Republic). After the production of the appropriate amount of the blend with melt compounding, using an internal mixer Brabender Plastograph at 190 °C as described previously, the blend was ground into small pieces using a grinder (Pulverisette 19 Universal Cutting from Fritsch, Idar-Oberstein, Germany) and introduced into a Filament Maker Composer from 3devo (Utrecht, The Netherlands), for the production of filaments at 190 °C.

Tensile and impact test specimens were printed with respect to the available standards (referenced standards used: for the tensile test samples, ISO527–1BA, and for the impact specimens, ASTM D256, and the little notch on the sample was made with the RAY-RAN 1900 notching apparatus (Ray-Ran Test Equipment Ltd., Nuneaton, UK)). For the printing process, a nozzle with a diameter of 0.4 mm was used. The main parameters used for the printing step (Prusa Slicer) were: printing temperature of 190 °C, extrusion wight, layer thickness, infill 100%, flow 15 mm^3^/s, printer speed 200 mm/s. It was also possible to print the successive layers while applying different printing angles. In this study, a crossed 45°/−45° raster direction angle was used, aiming at the generation of the most isotropic structures in terms of mechanical properties. A prototype of a 3D-printed prosthetic finger with different degrees of movement was also proposed, as an example of the potential application of the developed biomaterials.

### 2.3. Characterization

#### 2.3.1. Mechanical Characterization

Tensile tests were performed according to ASTM D638 Type V standard using an LR 10 K tensile bench from Lloyd Instruments (Bognor Regis, UK), with a deformation speed of 50 mm/min, a 0.5 N preload force, and a distance between grips set at 25.4 mm. All specimens were conditioned at 20 °C for 48 h before testing.

Impact strength was evaluated with a Ray-Ran 2500 pendulum impact tester, working on Notched Izod mode with sample dimensions of 63.5 × 12.7 × 3.2 mm^3^, following the ASTM D256 standard. Five specimens of each sample were previously conditioned at 20 °C for 48 h before testing.

#### 2.3.2. Thermal Characterization

A TA Instrument DSC Q200 (TA Instruments, New Castle, DE, USA) was employed under nitrogen flow for the thermal analysis of the different PHB/PCL-based blends. The samples were subjected to a first heating cycle at 10 °C.min^−1^ from −80 to 200 °C (120 °C for PCL), to get rid of their thermal history, before being cooled to −80 °C at 10 °C.min^−1^, followed by a second heating scan up to 200 °C. Thermogravimetric analysis (TGA) was carried out under nitrogen (80 mL.min^−1^), using a TGA II from Mettler Toledo (Columbus, OH, USA). The samples (10 mg) were placed into a 70 µL saphir sample holder and subjected to the first isothermal heating at 100 °C for removing moisture, and then subjected to a heating scan of 20 °C.min^−1^ up to 500 °C.

#### 2.3.3. Structural Characterization

The surface topology of the compressed samples was performed using AFM analysis, in a glove box at room temperature and with dry nitrogen, using the Bruker MultiMode 8-HR AFL setup (Billerica, MA, USA). For the characterization of the samples, commercially available PPPNCHR rectangular-shaped silicon probe (Nanosensors) was used. The cantilever’s normal spring constant (42 N.m^−1^) was defined by Sader’s method from the data on the resonance peak (230 kHz) and the planar dimensions of the probes. The acquisition of the micrographs was performed using Nanoscopes Analysis software (Version 1.8, Bruker) and the phase imagery was obtained in tapping mode. This mode allows one to overcome difficulties associated with friction, adhesion, electrostatic forces, and other occurring phenomena, placing the tip in contact with the surface to provide high resolution and then lifting the tip off the surface to avoid dragging the tip across the surface.

Wide angle X-ray diffraction analysis (WAXD) data were collected using a BRUKER D-5000 with Cu Kα radiation (λ = 1.5418 Å). The scattering angle (2Ө) domain studied ranged from 5° to 30° at 1 °min^−1^.

Volume variation of the different samples was measured in THF in order to evaluate the swelling ratio of the materials. The different specimens were first weighed prior to being immersed in tetrahydrofuran (THF) at room temperature. At regular intervals, samples were weighed after being wiped with Whatman filter paper. Volume variation (VR) was calculated by measuring the volume ratio (VR) between the final volume (Vf) and the initial volume (Vi) (Equation (1)):

Volume ratio = Vf/Vi(1)

## 3. Results

### 3.1. Properties of Non-Compatibilized PHB/PCL Blends

In the present study, different PHB-based blends were studied, as well their compatibilization. In the first part of the “Results and discussion” section, PHB/PCL materials are evaluated. Their compatibilization was enhanced by using DCP as a free-radical promoter, allowing grafting and cross-linking reactions, and low-molecular PEG was added as a plasticizer. For each of the studied systems, the thermal and physico-mechanical (tensile test and impact test) properties, as well as the morphological characteristics, were studied. As a final step, the 3D printing of the materials was performed, and a prototype of a prosthetic finger with several degrees of freedom was proposed.

As referenced materials, PHB and PCL (50,000 g.mol^−1^, see Materials Section) were first characterized for their physico-mechanical properties. The Young’s modulus values, stress at yield and at break, as well as strain at break and impact resistance are presented in Table 2. The obtained data for PHB, such as high stiffness (Young’s modulus of 1850 MPa and tensile strength of 42 MPa), low elongation at break values (4.7%), and low impact resistance (1.3 kJ.m^−2^), were in accordance with the results already published in the literature [9]. In contrast, PCL samples were characterized with expected elastomeric-like behaviour [19,20]: lower Young’s modulus (440 MPa) and higher elongation at break (570%), in comparison to PHB. As a further step, we explored the possibility of combining the polymers’ properties, while adjusting the weight ratio of PHB and PCL in the melt-processed blends. With this, we aimed to increase the PHB ductility and impact resistance. It is worth pointing out that such blends have been already investigated by Garcia et al. [18]. In the present study, the improvement in mechanical properties was not reached at the expected levels: PHB strain at break (3.3%) and impact resistance (1.23 kJ.m^−2^) in PHB/PCL (80/20) materials remained relatively low. Lead by the obtained results, PCL content was increased up to 40 wt.%. Once more, the elongation at break (2.7%) and the impact resistance (1.65 kJ.m^−2^) remained insufficient. Such difficulties were faced by Zytner et al. [24], where limited improvement in impact strength and elongation at break of PHB/PCL blends were observed in injection-molded materials. The decrease in the Young’s modulus values, induced by the presence of PCL, from 1840 MPa for the pristine PHB to 1200 MPa for the blend, remained consistent with the reported data [25,26].

**Figure 1:**
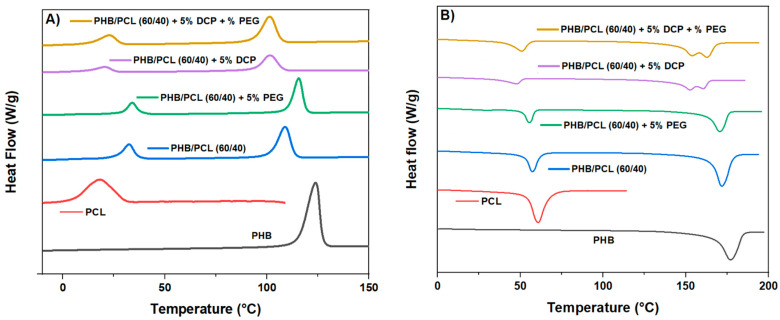
DSC thermograms of PHB, PCL and PHB/PCL blends: cooling step (**A**), second heating step (**B**).

As a next step, it was of importance to evaluate the materials’ thermal properties, as well as the degree of crystallinity of PHB and PCL, as this last parameter can greatly impact the mechanical behavior of the resulting materials. Therefore, DSC analysis was performed, and the thermograms of the materials and the related thermal transitions are presented in Figure 1 and Table 3. As can be seen, PHB and PCL proved mostly immiscible: in the case of PHB-PCL (60/40) blends, two endothermal individual peaks were observed at 171.0 °C and 57.3 °C, close in values to the typical Tm values for PHB (177.0 °C) and PCL (57.7 °C). The crystallinity degree of each of the polymers in the PHB-PCL (60/40) blend was slightly lower with respect to the values obtained for the neat polymers (from 58.3% to 57.0% for PHB) and from 38.2% to 33.0% in the case of PCL. This result clearly indicates that neither polymer presents a nucleating effect on the other one. In addition to this, a decrease in the PHB crystallization temperature (Tc) from 124 °C to 112 °C was recorded in the PHB-PCL (60/40) blend. This could be explained by the transfer of heterogeneities, that could act as nucleant agents, from PHB to PCL by retarding the primary nucleation [27]. Therefore, the presence of more heterogeneities in the PCL phase was responsible for the increase in PCL Tc from 20.2 °C up to 32.4 °C. The incorporation of PEG alone did not induce significant change in the PHB/PCL blend thermal behavior, since the Tc and the Tm, as well as the degree of the crystallinity remained close to those of PHB/PCL blend. This can be explained with the low rate (5 wt.%) of the plasticizer in the system.

**Figure 2:**
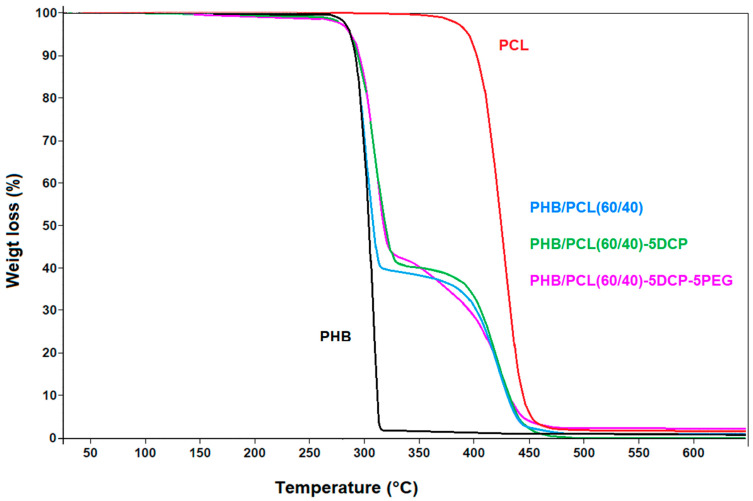
TGA thermograms of PHB, PCL and their blends (TGA analysis under N_2_).

The thermal degradation of the samples was studied with TGA analysis. As can be seen from the TGA thermograms (Figure 2) and the degradation temperature (Td) values (Table 4), PCL was characterized with higher thermal stability (Td of 425 °C and Td onset value at 5% of weight loss (Td-5%) of 395 °C), compared to PHB (Td of 300 °C and Td-5% 287 °C). Both polymers presented characteristic one-step thermal degradation.

In the case of the thermal analysis of PHB/PCL-based blends, two thermal transitions were observed, corresponding to the thermal degradation of each of the polymers (the first was assigned to the PHB, and the second to PCL as the more thermally stable one). The presence of two separated decomposition steps is typical for immiscible blends presenting phase segregation.

**Figure 3:**
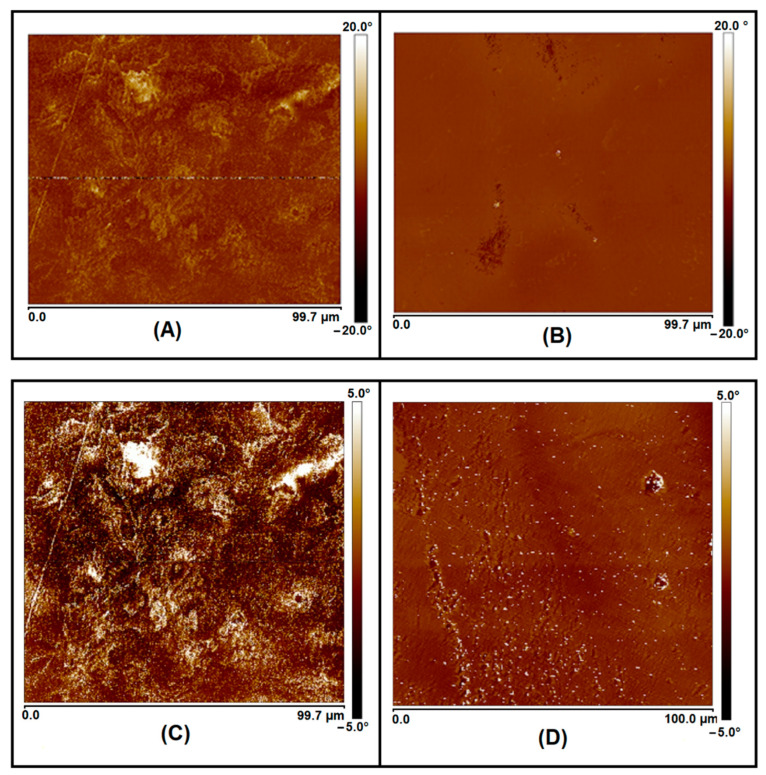
AFM micrographs of PHB/PCL (60/40) (**A**), PHB/PCL (60-40)-5DCP compatibilized blend (**B**), PHB/PCL (60-40)-5PEG (**C**) and PHB/PCL (60-40)-5DCP-5PEG (**D**).

For the better understanding of the materials’ properties, we proceeded with the morphological analysis of the blends with AFM. As presented in Figure 3A, an irregular morphological profile was observed for PHB/PCL (60/40). The clearer regions (yellowish domains) in the micrographs corresponded to rigid PHB-based domains, and the darker (brownish domains) to soft PCL-based domains of the sample. In this way, the presence of such domains explained the material’s mechanical properties that was found to be even more fragile (strain at break of 2.7%) than the pristine PHB (strain at break of 4.7%) (Table 2).

### 3.2. Effect of Reactive Melt Processing

**Figure 4:**
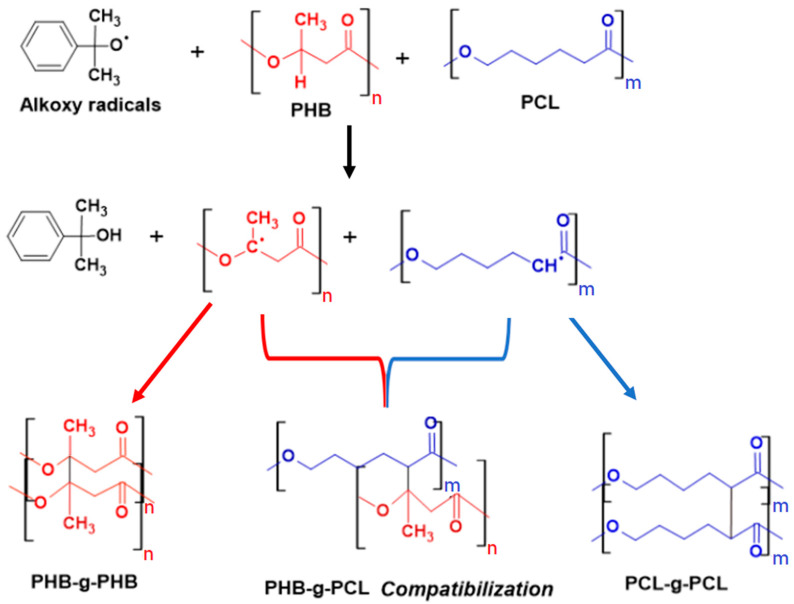
Schematic presentation of the possible grafting and (micro)crosslinking reactions occurring during the reactive melt process of PHB/PCL blends in presence of DCP.

Compatibilization is known as a suitable approach to enhance the mechanical performances of the materials in initially poorly miscible polymer blends. Both the morphology of the multiphase system and the ability of the interface to transmit stress from one phase to the other drive the mechanical properties of the blends [28,29]. This can be achieved by either the addition of compatibilizing agents (low molecular organic compounds or inorganic nanoparticles) or by promoting the formation of covalent bonds between the polymeric phases through in situ reactions during melt processing. For example, a coupling reaction between polyesters can be achieved by using dicumyl peroxide (DCP) as a free-radical initiator. DCP was used in initially immiscible polymeric matrices, e.g., different polyester binary blends such as PLA/PCL [30,31], PLA/PBS [32], PHB/PLDA [33], PHB/PBS [34] and PHB/PCL [35]. In fact, taking advantage of the thermal decomposition of DCP and the related O-O bond homolytic cleavage leading to the formation of cumyloxy radicals, it is possible to obtain new free-radicals along PHB and PCL chains with hydrogen abstraction that could occur either in the alpha position of the ester carbonyl (PCL and PHB) functions or on the tertiary carbon atom of PHB (Figure 4). PHB- and PCL-based free-radicals can be then combined at the interface to form PHB-g-PCL and PCL-g-PHB copolymers and even (partially) crosslinked PHB-PCL structures. Moreover, the free-radical combination reactions do not take place only at the interface of the polymer domains, but can also occur in PHB and PCL phases, thus inducing the formation of branched and crosslinked PHB and PCL. As a result, the final chemically complex material presents structurally pure PHB and PCL domains (branched, cross-linked (micro)networks, free macromolecular chains), in addition to the generated PHB-g-PCL and PCL-g-PHB copolymers formed at the domain interface. In Figure 4, a schematic representation of the possible grafting/cross-linking reactions involved in the case of PHB/PCL blends containing DCP is presented.

In the present study, the compatibilization of PHB/PCL by the incorporation of DCP (5 wt.%) led to a significant change in the material morphological characteristics, as determined by AFM analysis (Figure 3B). The homogenous phase was observed without the observation of visible PHB or PCL domains, providing evidence that the reactive melt processing enhanced the chemical affinity between the two initially immiscible PHB and PCL phases. Interestingly, this was directly validated by the materials’ mechanical characteristics: a clear decrease in the material Young’s modulus values (680 MPa), accompanied with a slight increase of the strain at break (5.1%), compared to the non-compatibilized blend (1225 MPa and 2.7%, respectively). In addition to this, the most important change was the clear increase in the impact resistance. For PHB/PCL (60-40)-5DCP blend, this parameter was 7.2 kJ.m^−2^, while it was below 2 kJ.m^−2^ for PHB and non-compatibilized PHB/PCL bend (60/40 or 80/20) (Table 2).

**Figure 5:**
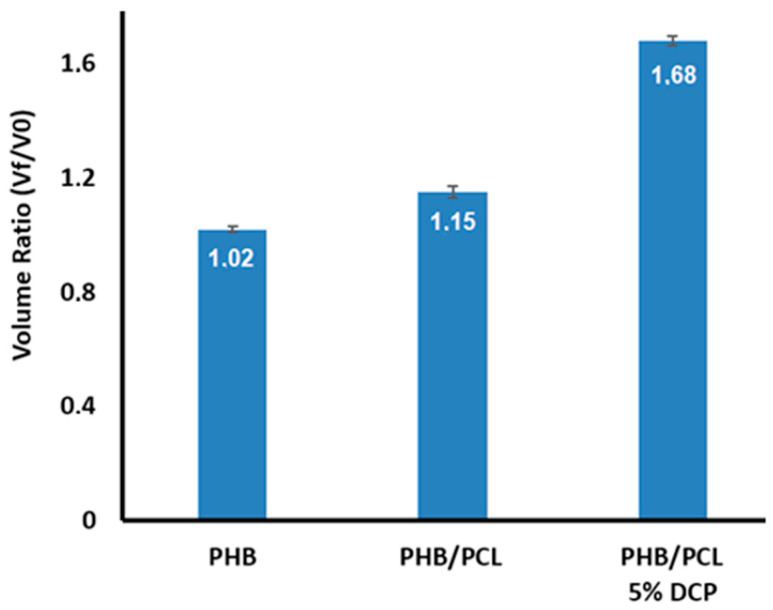
Volume ratio variation of PHB, PCL and PHC/PCL (60/40)-5DCP samples after 2 days of immersion in THF.

Additional information about the occurrence of the grafting/crosslinking reactions were obtained via swelling tests. For this step, PHB, PHB/PCL (60/40) and compatibilized PHB/PCL (60/40)-5DCP blend were immersed for 2 days in THF. As PHB is not soluble in THF, the swelling of the sample without weight loss will be the result of the grafting/crosslinking reaction between the two polymers and the solubilization or swelling of the PCL fraction in the final material. As expected, and confirmed from Figure 5, the PHB specimen did not undergo any volume change after 2 days of immersion. In the case of PHB-PCL (60/40), only a slight volume increase was noticed (volume ration 1.15). Despite the presence of 40 wt.% of PCL, and the unchanged weight mass of the samples, we explained this result with the entrapment of PCL in the non-soluble PHB-matrix (AFM analysis). However, in the case of the PHB-PCL (60/40)-5DCP, a significant swelling of the material was obtained (an increase in the volume ratio up to 1.68) without further loss in the sample integrity, confirming the grafting/crosslinking structure. With it was confirmed the formation of the successful grafting reaction thought the incorporation of DCP and the generation of the partially crosslinked structure. The proposed reactive melt processing of the PHB/PCL-based blends, using high DCP content (5 wt.%), led to the formation of a partially crosslinked PHB-PCL copolymer structure, accompanied by: (i) blend morphology refinement (AFM analysis), (ii) enhancement of the materials’ mechanical properties (significant increase of the impact resistance up to 7.2 kJ.m^−2^) and physico-chemical affinity (organic solvent THF), as well as (iii) fine-tuning of the materials’ thermal transitions and the degree of crystallization.

The thermal properties and degree of crystallinity of the new structure were also studied using DSC analysis. Unlike the PHB/PCL (60/40) blend, where both polymers preserved their thermal characteristics and crystallization properties, the use of DCP induced significant modifications. First, the Tm of both polymers decreased (confirming once more the compatibilization of PHB and PCL), and an additional PHB melting peak was noticed. Indeed, in the compatibilized blend, PHB had a double melting peak at 153 °C and 162 °C (in the PHB/PCL (60-40) blend, PHB was characterized with a single melting peak at 171 °C). Double melting behavior can be induced either by a melting and recrystallization of the crystalline domains [36] or by the formation of spherulites of different lamellar thicknesses [37,38]. As far as PHB is concerned, previous works have reported its double-melting behavior that occurs according to the melting and recrystallization model [39,40]. Thus, the presence of the two melting peaks indicated significant recrystallization during melting. The low-temperature melting peak corresponds to metastable crystals while the high-temperature peak is related to the melting of ordered crystals [41]. The presence of such metastable as-formed crystals can be attributed to the grafting process that induces some variations in PHB chain mobility, between grafted (macromolecular chains with low mobility) and unmodified linear polymer chains.

The decrease in the PHB Tc, already observed in the PHB/PCL (60-40) blend, and which was attributed to the migration of heterogeneities from PHB to the PCL phase, was further accentuated in the case of the reactive blend. In fact, this temperature decreased from 112 °C in the case of the PHB/PCL (60-40) blend to 98 °C when DCP was added. This was an indication that the PHB crystallization process became more difficult. This phenomenon was explained with the presence of the new chemical structure (grafted and partially crosslinked copolymer). Moreover, the addition of 5 wt.% DCP induced some reduction of both PHB and PCL degrees of crystallinity, in comparison with their degrees of crystallinity in the non-compatibilized blend: from 57% to 26% for PHB and from 33% to 27% for PCL. Such evolution has been already reported previously in the case of PHB/PCL-DCP (DCP 1 wt.%) [18].

**Figure 6:**
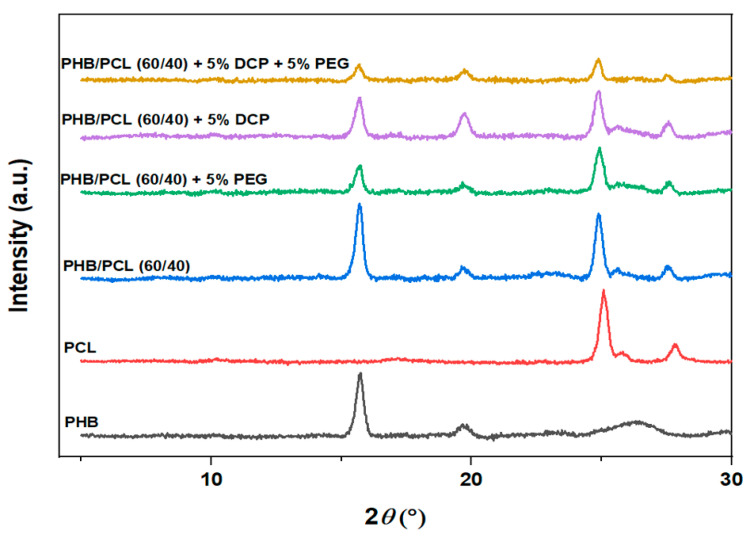
Diffractograms of PHB and PCL materials containing PEG and/or DCP.

The nature of the crystalline phases of both polymers was not affected either by the presence of the other polymer nor by the reactive compatibility with the DCP. Indeed, each polymeric phase in the blend crystallized in the same crystalline forms as in the separated polymers, as evidenced by XRD analysis (Figure 6). XRD diffractograms of the blends are very similar, whatever their composition, and correspond to the superposition of PHB and PCL typical peaks.

The reactive blends were studied using TGA, and in all cases the reactive compatibilized blends presented only a slight increase of the thermal stability during the first degradation step than PHB (Figure 2 and Table 4). Indeed, it has been reported that the compatibilization of immiscible polymer blends, such as polypropylene and polyamide, induces an improvement of the thermal stability of the less thermal-resistant phase [42].

### 3.3. Effect of Combining Reactive Melt Processing and Plasticization

In the literature, the use of suitable plasticizers, such as PEG, polypropylene glycol or triethyl citrate, has been already demonstrated to enhance PHA-based materials’ mechanical properties [43,44]. In our study, PEG was selected as a low-molecular-weight plasticizer for the compatibilized PHB/PCL (60/40)-5DCP blend. To prepare the complex system, firstly the PHB/PCL (60/40) and the appropriate amount of DCP were melt-blended (using a Brabender^®^ internal mixer), and then the plasticizer was added. To have a better comprehension of the plasticizer effect, PEG was also incorporated into a non-compatibilized PHB/PCL (60/40)-5PEG blend.

The mechanical properties of the new materials revealed that Young’s modulus, stress at break, strain at break and impact resistance remained close in values to those of non-compatibilized PHB/PCL blends (Table 2). The morphology of the two blends, i.e., PHB/PCL (60/40)-5PEG and PHB/PCL (60/40), evaluated by AFM, remains very similar: both present phase separation morphologies (Figure 3).

However, the effect of the use of the plasticizer was more pronounced in the compatibilized blend PHB/PCL-DCP: an important increase in the impact resistance from 7.2 kJ.m^−2^ for PHB/PCL (60/40)-5DCP to 15.4 kJ.m^−2^ for PHB/PCL (60/40)-5DCP-5PEG was obtained, while it was only about 1.5 kJ.m^−2^ for the PHB/PCL-5PEG blend. The incorporation of the plasticizer does not induce any positive effect on the blend’s mechanical properties, contrary to what was already reported in the literature [44]. The ineffectiveness of the plasticizer in our case is explained by its low incorporation rate (only 5 wt.%), whereas it is usually incorporated at a much higher rate, i.e., min. 20 wt.%.

**Figure 7:**
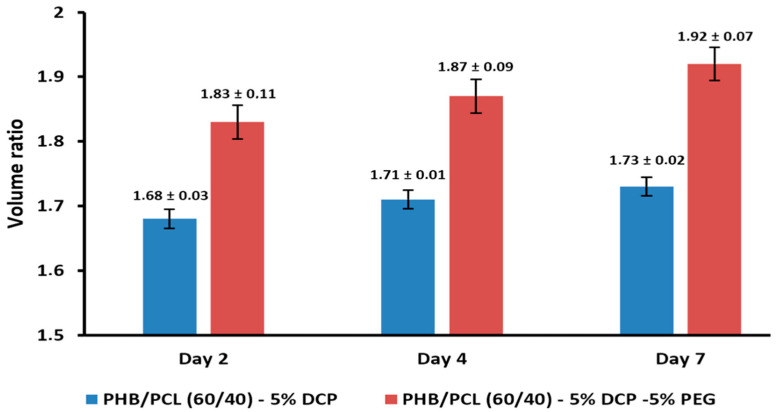
Effect of the incorporation of PEG on the specimen volume ratio, immersed for 2, 4 and 7 days in THF.

Even at low incorporation content, the incorporation of 5 wt.% PEG in compatibilized blends induces a significant change in the blend’s physico-chemical behavior as evidenced by the evolution of the swelling properties (Figure 7). After 2 days of immersion, the volume ratio for PHB/PCL (60/40)-5DCP-5PEG increased to 1.83, and this effect was even more pronounced after 7 days of immersion (volume ratio up to 1.93, or 20% of additional volume ratio increase).

**Figure 8:**
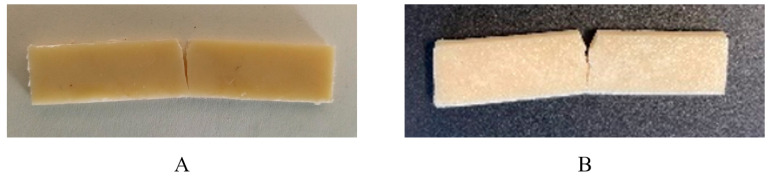
Partial fracture of the thermal compressed PHB/PCL (60/40)-5DCP-5PEG specimen (63.5 × 12.7 × 3.2 mm^3^, ASTM D256 standard) after impact test (**A**) and of the 3D printed PHB/PCL (60/40)-5DCP-5PEG samples (PCL 80 kDa) (**B**).

It is worth mentioning that, in contrast to the other compositions, PHB/PCL (60/40)-5DCP-5PEG specimens did not break completely during the impact test (Figure 8). These results are very important and show that the enhancement of the mechanical properties of the PHB/PCL blend was not induced by the plasticizer (i.e., PEG) but by the combination of the reactive process, via the incorporation of DCP together with the plasticizer addition.

DCP is typically used in reactive extrusion and PHB/PCL blends at 1 wt.% content [18]. In order to evaluate the effect of DCP content on blends mechanical properties, additional PHB/PCL (60/40)-1DCP-5PEG specimens were prepared respecting the same working conditions. As can be observed in Table 2, the reduction of DCP content, from 5 to 1 wt.%, induces an important decrease of the impact resistance that decreased from 15.4 kJ.m^−2^ to 6 kJ.m^−2^).

As a final step in this study, we explored the printability of the obtained materials using the fused deposition modelling (FDM) 3D printing technique. For this, custom-made continuous filaments with appropriate diameter (1.75 mm) were produced from the synthesized materials. In the literature, it is well described that polymer materials printed by FDM present anisotropic mechanical properties mainly impacted by the deposition direction of the filaments in the adjacent layers [45]. After fine-tuning the printing conditions, impact and tensile test specimens were produced with dimensions referencing standards (tensile tests, ISO527–1BA; impact tests, ASTM D256). Here, we made the choice to print the impact and the tensile specimens in −45°/45° configuration with respect to the XYZ axes references of the Prusa FDM printer, described today as the most promising for the fabrication of printed specimens with a lower degree of anisotropy. The most interesting mechanically performant PHB/PCL blend, i.e., PHB/PCL (60/40)-5DCP-5PEG (PCL of 50 kDa), was used for the production of filaments by extrusion and 3D printing of specimens for tensile and impact tests. The obtained data are presented in Table 5. The blend, thus containing PCL with 50 kDa molecular weight, presented lower impact resistance (3.18 kJ.m^−^^2^) than the corresponding compressed molded specimens (15.4 kJ.m^−^^2^). To better understand this result, a second composition with another PCL sample, displaying a higher-molecular-weight PCL (80 kDa), was prepared and tested. This new 3D-printed composition was characterized with enhanced impact resistance (7.2 kJ.m^−^^2^). Moreover, the specimen containing PCL 80 kDa presented a partial fracture, in contrast to the specimens with PCL 50 kDa that was completely broken (Table 5). The positive effect obtained by increasing PCL molecular weight was not present in the compressed molded specimens (Table 5). In fact, the impact resistance of this new composition, containing PCL with a higher molecular weight and prepared by compression molding, remained high and similar to the blend containing PCL 50 kDa. This result highlighted the fact that the polymer molecular weight and the degree of the macromolecular entanglements highly impact the possibility of producing stable and uniform filaments suitable for 3D printing.

**Figure 9:**
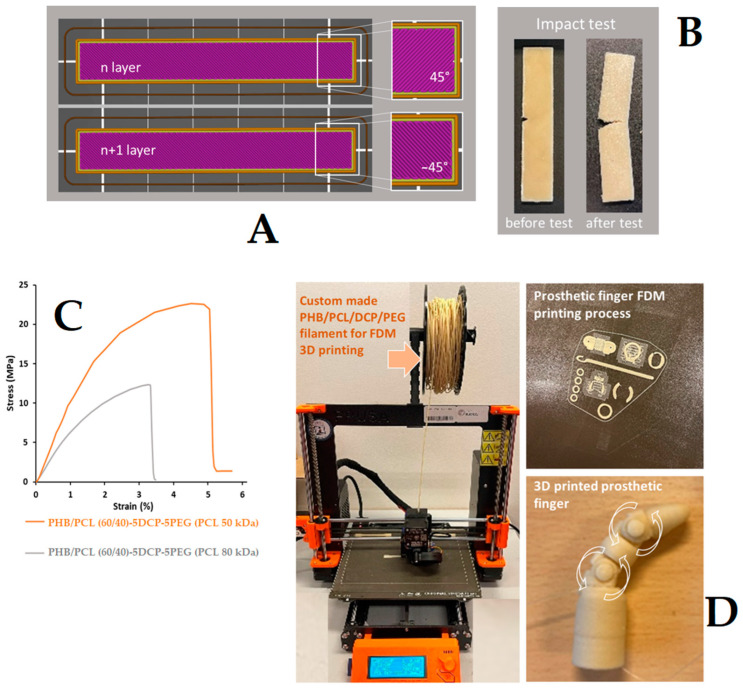
PHB/PCL-based materials FDM 3D printing: −45°/45° filament deposition during the printing process of impact test samples (**A**). Sample images before and after impact tests of blend containing PCL (80 kDa) (**B**), and stress–strain curves of the printed materials (**C**). FDM 3D printed prototype presenting prosthetic finger with different degrees of freedom (**D**). Specimens presented in images (**A**,**B**) present same size 63.5 × 12.7 × 3.2 mm^3^ (ASTM D256, standard).

The tensile properties of the printed samples are presented in Figure 9. It can be observed that the Young’s modulus of the PHB/PCL (60/40)-5DCP-5PEG (PCL 50 kDa) was higher (1050 MPa) than the materials containing PCL 80 kDa (645 MPa). In comparison, the Young’s modulus value obtained with PCL80 in the PHB/PCL (60/40)-5DCP-5PEG composition prepared by 3D printing proved similar to what was recorded in compression molding of the blend with the same composition but with PCL 50. It is important to notice that the degree of PHB and PCL crystallinity in specimens obtained with 3D printing were lower compared to those obtained by compression molding (Table 3 and Table 6). With this finding it was evidenced that it is of importance to consider the processing approach in the selection and fabrication of the polymer materials. A prototype of a prosthetic finger was also produced, highlighting the great potential of the developed polymer systems (Figure 9D). The degree of mobility of the resulting materials provides additional added value to the proposed polymer systems offering application as biomaterials.

## 4. Conclusions

In the present study, a series of PHB/PCL-based materials were obtained using reactive melt-mixing, aiming to improve the mechanical properties of PHB. For this purpose, DCP was used as a compatibilizing promotor and grafting agent in the initially poorly miscible PHB/PCL blends, while PEG was added as a low molecular plasticizer. The successful production of PHB/PCL (60/40)-5DCP led to an important refinement of the blend morphology, a slight increase in the elongation at break and a significant improvement in the impact resistance (7.2 kJ.m^−2^) of the materials, as compared to PHB (1.3 kJ.m^−2^). This was also accompanied with a slight improvement of the thermal stability of the compatibilized blend and PHB melting endotherm (bimodal with maximal pack values of 154 °C and 162 °C), revealing the occurrence of a new chemical structure based on PHB and PCL (through the formation of grafting and crosslinking reactions). Finally, the addition of low molecular PEG as a plasticizer resulted in the production of PHB/PCL (60/40)-5DCP-5PEG materials reaching even higher impact resistance values (15.4 kJ.m^−2^). In accordance with the high impact resistance of the final materials, it was found that the degree of crystallinity was decreased in the complex system, thus confirming the positive impact of the proposed approach. PHB/PCL (60/40)-5DCP-5PEG composition presents the advantage of containing higher biobased content and superior impact resistance than elastomeric petro-sourced PCL. This work presents an additional step towards the development of efficient PHB-based materials that can be a subject of forthcoming studies dealing with the incorporation of reinforcing nanofillers for enhancing a material’s elastic modulus, while preserving their high impact resistance, owing to their low incorporation rate.

Through this approach, it was possible to move one step closer to the melt processing and fabrication of materials with complex shapes and designs, as demonstrated by the proposed FDM 3D-printed prosthetic finger prototype, revealing the potential of next-generation PHB-based biomaterials. This type of blend would be very suitable for external prostheses prepared by 3D printing, which could therefore be composted after use.

## Figures and Tables

**Table 1:** Blend compositions.

Sample	PHB (wt.%)	PCL (wt.%)	DCP (wt.%)	PEG (wt.%)
PHB	100	0	0	0
PCL	0	100	0	0
PHB/PCL (80/20)	80	20	0	0
PHB/PCL (60/40)	60	40	0	0
PHB/PCL (60/40)-5DCP	57	38	5	0
PHB/PCL (60/40)-5PEG	57	38	0	5
PHB/PCL (60/40)-5DCP-5PEG	54	36	5	5
PHB/PCL (60/40)-1DCP-5PEG	56.4	37.6	1	5

**Table 2:** Tensile results of PHB/PCL materials containing DCP and PEG.

Sample	Young’s Modulus (MPa)	Stress at Break (MPa)	Strain at Break (%)	Impact Resistance (kJ.m^−2^)
PHB	1840 ± 115	42 ± 2.4	4.7 ± 0.3	1.3 ± 0.1
PCL	440 ± 34	33.0 ± 2	570 ± 30	4.6 ± 0.7
PHB/PCL (80/20)	1430 ± 110	34.5 ± 2.9	3.3 ± 0.2	1.3 ± 0.3
PHB/PCL (60/40)	1225 ± 40	19.3 ± 0.4	2.7 ± 0.5	1.7 ± 0.2
PHB/PCL (60/40)-5DCP	680 ± 70	19.1 ± 20	5.1 ± 0.4	7.2 ± 1.8
PHB/PCL (60/40)-5PEG	1060 ± 90	17.0 ± 1.6	1.8 ± 0.2	1.5 ± 0.4
PHB/PCL (60/40)-5DCP-5PEG	640 ± 5	18.6 ± 2	5.5 ± 1	15.4 ± 1.5
PHB/PCL (60/40)-1DCP-5PEG	890 ± 20	21.5 ± 0.1	4 ± 0.1	6 ± 0.5

**Table 3:** DSC data of PHB, PCL and PHB/PCL-based materials.

Sample	Tm (°C) (2nd Heating)		Tc (°C)		Degree of Crystallinity (%)	
	PHB	PCL	PHB	PCL	PHB	PCL
PHB	177.0	n.a.	124.0	n.a.	58.3	n.a.
PCL	n.a.	57.5	n.a.	20.2	n.a.	38.2
PHB/PCL (60/40)	171.0	57.3	112.0	32.4	57.0	33.0
PHB/PCL (60-40)-5PEG	172.0	63.0	115.0	34.0	64.0	33.0
PHB/PCL (60/40)-5DCP	153.5; 162.0	48	98.0	20.4	52.8	27.0
PHB/PCL (60/40)-5DCP-5PEG	153.6; 163.0	51.0	101.3	22.8	54.0	35.5

**Table 4:** Degradation temperature of PHB, PCL, and PHB/PCL blends.

Sample	Td_-5%_ (°C)	T_d_ PHB (°C)	T_d_ PCL (°C)
PHB	287	303	---
PCL	395	---	425
PHB/PCL (60/40)	287	300	420
PHB/PCL (60/40)-5DCP	290	313	422
PHB/PCL (60/40)-5DCP-5PEG	288	317	416
PHB/PCL (60/40)-5PEG	268	291	417

**Table 5:** Comparison of the effect of PCL molecular weight on the 3D-printed and compression-molded specimens’ impact resistance.

Sample	Impact Resistance (kJ.m^−2^)	Fracture
3D-printed samples		
PHB/PCL (60/40)-5DCP-5PEG (PCL 50 kDa)	3.18 ± 0.3	Total
PHB/PCL (60/40)-5DCP-5PEG (PCL 80 kDa)	7.23 ± 1.5	Partial
Compression-molded samples		
PHB/PCL (60/40)-5DCP-5PEG (PCL 50 kDa)	15.4 ± 1.5	Partial
PHB/PCL (60/40)-5DCP-5PEG (PCL 80 kDa)	14.2 ± 1.2	Partial

**Table 6:** Printed samples tensile test results.

Sample	Young’s Modulus (MPa)	Stress at Break (MPa)	Strain at Break (%)	χc PHB/χc PCL (%)
PHB/PCL (60/40)-5DCP-5PEG (PCL 50 kDa)	1046.6 ± 65.6	21.3 ± 0.7	5.1 ± 0.1	32/14
PHB/PCL (60/40)-5DCP-5PEG (PCL 80 kDa)	642.5 ± 85.4	12.1 ± 0.9	3.5 ± 0.4	33/18

## Data Availability

Not applicable.
